# Prosthetic Outcomes in Stage III/IV Periodontitis Patients With 20–29 Years of Supportive Periodontal Care

**DOI:** 10.1111/jcpe.70036

**Published:** 2025-09-15

**Authors:** T. Eger, F. Wörner, N. Lingwal, P. Eickholz

**Affiliations:** ^1^ Department of Dentistry l Periodontology Bundeswehr Central Hospital Koblenz Koblenz Germany; ^2^ Center for Dentistry and Oral Medicine (Carolinum) Goethe‐University Frankfurt am Main Frankfurt am Main Germany; ^3^ Department of Periodontology Center for Dentistry and Oral Medicine (Carolinum), Goethe University Frankfurt am Main Frankfurt am Main Germany

**Keywords:** long‐term supportive periodontal care, partial fixed or removable dentures, periodontal therapy, periodontitis, tooth loss

## Abstract

**Objective:**

To assess (i) tooth loss (TL) in treated periodontitis (stage III/IV) patients undergoing supportive periodontal care without or with partial denture (PD) (removable [RPD], fixed PD [FPD], implant supported FPD [iFPD]) and (ii) 10‐year prosthetic outcomes.

**Methods:**

From 1993 to 2023, comprehensive periodontal therapy including restorative treatment was rendered at the Bundeswehr Central Hospital periodontology outpatient clinic.

**Results:**

Two‐hundred and thirty‐three patients with generalised stage III/IV periodontitis were treated with a mean follow‐up of 21.7 ± 2.7 years: 12 with 21 clasp‐retained metal‐based RPDs (TL/year 0.4 ± 0.39), 12 with 20 double crown–retained metal‐based RPDs (0.35 ± 0.42), 50 with 80 ≥ 5‐unit FPD (0.23 ± 0.33) and 27 with 218 implants (0.15 ± 0.22). One‐hundred and thirty‐two patients with < 5‐unit FPDs, no RPDs or iFPDs served as control. The control group exhibited the lowest rate of TL (0.05 ± 0.08). Multivariate analyses revealed age (*p* < 0.001), smoking > 10 cigarettes/day (*p* < 0.001) and RPD (*p* = 0.002)/FPD (*p* < 0.001) but not iFPD (*p* = 0.134) to be significantly associated with TL. After 10 years, in 75% patients 67% of the metal‐based clasp‐retained RPDs; in 80% of patients 75% of double crown RPDs; in 90% of patients 93% of FPD; and in 76% of patients 83% of iFPDs were functional.

**Conclusion:**

Stage III/IV periodontitis patients treated with RPD and FPD have a higher long‐term TL rate than controls and iFPD.

## 1 Introduction

Periodontitis is the inflammatory progressive destruction of the periodontium triggered by a dysbiotic biofilm. The main features of periodontitis are clinical attachment loss, radiographic bone loss, periodontal pockets and bleeding (Papapanou et al. 2018). Periodontitis is further classified in two dimensions: stage/extent and degree. Stage captures disease severity and complexity of treatment; grade additionally captures the observed and/or expected rate of progression (Papapanou et al. 2018; Tonetti et al. 2018).

Tooth loss (TL) is a true clinical endpoint of clinical studies in dentistry. According to the 2018 Classification, stage IV periodontitis is identified from the larger population of individuals with stage III periodontitis (Herrera et al. 2022). Stage IV is defined as TL due to periodontal reasons of ≥ 5 teeth and/or stage III plus the need for complex rehabilitation due to the presence of one or more of the following factors (Papapanou et al. 2018; Herrera et al. 2022):

- tooth hypermobility due to secondary occlusal trauma that can be corrected without tooth replacement (case type 1);
- pathological tooth migration, characterised by tooth elongation, drifting and flaring, which is amenable to orthodontic correction (case type 2);
- partial edentulism that can be prosthetically restored without full‐arch rehabilitation (case type 3);
- partial edentulism that needs to be restored by means of full‐arch rehabilitation, either tooth‐ or implant‐retained, and either fixed or removable (case type 4).

In periodontitis stage IV type 4 patients with an insufficient number/distribution of periodontally maintainable teeth to support a full‐arch fixed partial denture (FPD), a tooth‐supported full‐arch removable partial denture (RPD) may be considered (Donos et al. 2022). Rehabilitation of partial edentulism with implant‐supported prostheses enhances the quality of life (Gennai et al. 2022). However, substantial evidence indicates that subjects with advanced/rapidly progressing forms of periodontitis have a higher risk of peri‐implantitis and implant loss when compared with the general population or with individuals without a history of periodontitis (Schwarz et al. 2018; Carral et al. 2021; Carra et al. 2022).

This retrospective study aimed to determine (i) TL in patients without and with FPD, implant supported FPD (iFPD) and RPD after ≥ 20 years of supportive periodontal care (SPC), and (ii) the 10‐year survival rates of the various partial dentures (PDs) in patients with treated generalised stage III/IV periodontitis.

## 2 Material and Methods

### 2.1 Patients

All patients referred by their military dentists for specialist treatment to Department XXIII Dentistry‐Periodontology, Central Hospital of the German Armed Forces, Koblenz, Germany, and underwent comprehensive periodontal therapy were screened for this cohort study. These treatments took place between 1993 and 2023. The cohort analysed in this study is a subgroup of a cohort reported earlier (Eger et al. 2013).

The present study was based on a questionnaire to record stress and smoking behaviour at baseline, after accomplishment of APT, prior to prosthodontic treatment and after 20 years of SPC. All participants were informed about the study and gave written informed consent. This cohort was analysed according to prosthodontic treatment after APT: no prosthodontic treatment (control) or PD. To control for additional diseases having an unfavourable effect on periodontal treatment, the patients were asked to undergo a blood test to identify diabetes, HIV infection and leukaemia and to detect inflammatory parameters by a blood count by their military doctor at their home base. According to their self‐reported smoking history, patients were categorised as former smokers (patients who had quit smoking before treatment) or non‐smokers (patients who had never smoked in their lives). All other patients were classified as current smokers. Smokers were grouped into moderate smokers (S1: ≤ 10 cigarettes per day) and heavy smokers (S2: > 10 cigarettes per day).

Patients were categorised according to their socioeconomic status using military rank and education level:

1. up to master sergeant/middle school plus professional training;
2. first sergeant to sergeant major/middle school plus professional training and specialised independent position;
3. subaltern officer/high school;
4. staff officer/university education.

It was documented whether and how often patients had received SPC and whether they had regularly adhered to the recommended SPC intervals. Patients attending 80% of all SPC visits within the recommended time of 1 month were designated as fully adherent (Demetriou et al. 1995). Patients attending 50%–80% of all SPC visits and extending the recommended intervals by 1 month but not more than 100% were accounted for as partially adherent. If patients had extended the recommended SPC interval at least once by over 100%, they were accounted for as non‐adherent (Eickholz et al. 2008) and were excluded from the study. Retrospectively, each patient was assigned a baseline diagnosis of periodontitis generalised stage III or IV according to the 2018 Classification of Periodontal Diseases (Papapanou et al. 2018; Billings et al. 2018; Tonetti et al. 2018).

Initial assessment encompassed medical and dental history and documentation of clinical dental findings, as well as periodontal charting using a manual, rigid periodontal probe (periodontal probing depths [PPD], clinical attachment level, bleeding on probing [BOP] at six sites per tooth [PCPUNC15, Hu Friedy, Chicago, USA], furcation involvement; Hamp et al. 1975; [PQ2N, Hu Friedy] and tooth mobility; Lindhe and Nyman 1977). All patients underwent comprehensive periodontal therapy with motivational advice for effective oral hygiene (step 1), non‐surgical periodontal therapy as ‘full‐mouth deep scaling and root planing’ (FMDSRP) using sonic scalers and hand instruments in one session under infiltration anaesthesia (step 2) and periodontal re‐examination including a follow‐up cleaning after 6 weeks. In case of any indication for concomitant systemic antibiotic treatment (Sanz et al. 2020), this was carried out with amoxicillin (3 × 500 mg/day) and metronidazole (3 × 400 mg/day) for 1 week (*n* = 142). As adjunct to the subsequent oral hygiene, patients applied 1% chlorhexidine gel (Chlorhexamed Mundgel, GSK, Munich) at home twice daily for 2 weeks. In teeth exhibiting persisting pockets with PPD ≥ 6 mm, periodontal surgery was recommended.

Six to 12 weeks after accomplishment of active periodontal therapy (APT: non‐surgical and if appropriate surgical periodontal therapy), all periodontal findings were re‐evaluated. If PPD ≥ 6 mm with BOP or suppuration after probing persisted, systemic antibiotics and, if necessary, further periodontal surgery were administered until there were no bleeding sites with PPD ≥ 6 mm. For all patients not requiring prosthodontic treatment (control group), this time point was taken as baseline (BL) for the assessment of TL.

SPC visits included assessment of approximal plaque index (API) (Lange et al. 1977), re‐instruction and re‐motivation to achieve effective plaque control and professional mechanical plaque removal with hand instruments followed by polishing of all teeth using rubber cups and polishing paste followed by the application of a fluoride gel. Dental status and PPD were obtained at six sites per tooth semi‐annually. BOP was recorded 30 s after probing. Sites exhibiting PPD = 4 mm and BOP and sites with PPD ≥ 5 mm were subjected to subgingival instrumentation (Eickholz et al. 2008). SPC intervals were assigned according to individual periodontal risk assessment criteria (every 3, 6 or 12 months) (Eickholz et al. 2008).

### 2.2 Prosthetic Restorations

In all patients except those in the control group, prosthodontic treatment was required, which was performed after APT. The association of prostheses with TL in periodontally compromised but treated patients was evaluated. The observation period started with the insertion of the first PD as specified below (baseline).

The list of periodontal treatments and order books for PD of the Department XXIII Dentistry‐Periodontology German Armed Forces Central Hospital, Koblenz, were screened for qualifying patients to form the following five groups:

- without major PD (FPD with < 5 units, no RPD, ≤ 2 implants) after APT (control group);
- FPDs: bridges with ≥ 5 units (abutments and pontics) and supported exclusively by teeth;
- metal‐based RPDs retained with clasps (RPDCs);
- metal‐based RPDs retained with double crowns (telescopic attachments) (RPDDs);
- FPDs on implants (iFPDs).

Individuals with different types of prostheses in different jaws (e.g., FPD in the maxilla and RPDD in the mandible) were assigned to the group of the major PD that was inserted first. Patients with major PDs that were already in place before periodontal therapy were excluded.

This was done to enrol as many qualifying patients as possible and to include at least 14 patients per group, because this group size was used in previous studies on periodontitis patients with different prosthodontic treatment options (Muller et al. 2013; Fardal and Linden 2008).

### 2.3 Statistical Analysis

For this retrospective cohort study, the patient was considered as the statistical unit. The primary outcome variable of this study was TL after APT with or without prosthodontic rehabilitation. It was assessed by comparing the number of teeth at the first and last (20–29 years later) SPC. Third molars were excluded from the analysis. The secondary outcome variable is the percentage of prosthodontic reconstructions inserted after APT in function for at least 10 years.

Descriptive data were presented as absolute or relative frequencies and mean ± standard deviations. Frequencies (gender, smoking status, socioeconomic status, diabetes mellitus) and mean ± standard deviations (age, observation period, TL) were calculated and compared between control and prosthetic groups as well as the four prosthetic subgroups (control group, FPD, iFPD, RPDC, RPDD) using Chi‐square tests or *t*‐tests/univariate ANOVA tests. For the identification of putative influencing factors, frequencies and mean ± standard deviations of subgroups (stage III/IV, diabetes mellitus [yes/no], fully/partially adherend, current smoker/non‐smoker and former smoker) were compared using Chi‐square tests or *t*‐tests/univariate ANOVA tests. Significance levels were not adjusted for multiple testing. Therefore, *p* values < 0.001 were considered significant.

Patient data were analysed to identify factors influencing tooth loss. Poisson regression models were initially applied, incorporating an offset term to account for varying baseline tooth counts. Because of the observed overdispersion, negative binomial regression models were subsequently fitted to better accommodate the data. All analyses were performed using R software (version 4.2.3). After providing significant inter‐group differences of *p* < 0.15 for univariate comparison, the following independent variables were entered into the models: control versus any type of major PD (model 1)/control (reference) versus RPDC versus RPDD versus FPD versus iFPD (model 2), gender, age (1‐year increment), adherence to SPC (fully/partially), years of SPC, smoking (non‐smoker [reference] vs. former smoker, S1, S2) and education level. Using logistic regression, associations between the dependent variable, namely survival of PD after 10 years, and the following independent variables were tested: any type of prostheses (FPD, iFPD, RPDC, RPDD), age, gender, compliance to SPC (fully [1]/partially adherent [0]), number of SPC after insertion of PD, diabetes mellitus and smoking (non‐smoker [0], former smoker [1], ≤ 10 cigarettes/day [2], > 10 cigarettes/day [3]). For logistic regression, always the PD that was inserted first was included and, thus, determined the PD type.

Statistical analyses were performed using PC programs (SystatTM for Windows Version 13, Systat Inc. Evanston, IL, USA; R version 4.2.3).

## 3 Results

Two‐hundred and thirty‐three periodontitis patients (49 females/21%; 173 periodontitis stage III/74.2%, 60 stage IV/25.8%) voluntarily participated. A total of 574 teeth (10.6%; annual loss of 0.13 ± 0.24) were lost in all patients over the study period (SPC/SPC after insertion of restorations) of 10–29 years (21.7 ± 2.7). Patients with stage III periodontitis lost 339 teeth (7.7%; annual loss of 0.11 ± 0.23 teeth) over the study period of 10–29 years (22.2 ± 4.5). Patients with stage IV periodontitis lost 235 teeth (23.0%; annual tooth loss of 0.22 ± 0.27 teeth) over the study period of 11–29 years (20.2 ± 5.0) (*p* = 0.001).

The following major PDs were inserted after APT between 1993 and 2013: in 12 patients 23 RPDCs, in 12 patients 20 RPDDs, in 61 patients 80 ≥ 5‐pontic FPDs and in 50 patients 218 implants. The following combination treatments were carried out: 16 patients with FPD + iFPD, 4 patients with RPDD + iFPD, 3 patients with RPDC + RPDD + FPD, 3 patients with RPDC + iFPD, 3 patients with RPDD + iFPD and 1 patient each with RPDC + FPD + iFPD, RPDC + FPD, RPDC + RPDD and RPDD + FPD + iFPD.

The control group (no/no major PD; *n* = 132) showed the lowest TL during the 23.7 years period with an annual loss rate of 0.05 teeth/year (Table 1). In total, more teeth were lost in the PD group than in the control and implant groups (Tables 1 and 2) and between the different FPDs and RPDs (Table 2). Patients with PD (45.8 ± 7.7 years) were older than those in the control group at baseline (42.6 ± 10.3 years; *p* = 0.016). Patients with PD were more often smokers than those in the control group (Table 1; *p* < 0.001), and patients without major PD and FPD had a higher number of teeth at baseline (Table 2).

**TABLE 1:** Characteristics of patients without (control) and with major restorations (bold: *p* < 0.001).

Major restorations	Without	With	*p*
Number of patients	132	101	
Men (*n*/%)	105/79	79/78	0.513
Age at BL (years)	42.6 ± 10.3	45.8 ± 7.7	0.016
Smoking BL (*n*/%)			
Non‐smokers	56/42	25/25	**< 0.001**
Former smokers	25/19	19/19	
1–10 cigarettes/day	33/25	20/20	
> 10 cigarettes/day	18/14	37/37	
Diabetes (*n*/%)	16/11	13/13	0.864
School education at BL (*n*/%)			
Middle school	1/1	3/3	0.088
Middle school +2	35/27	25/25	
High school	35/27	39/39	
Post‐secondary education	61/45	34/34	
Periodontitis stage III (*n*/%)	129/98	44/44	**< 0.001**
Periodontitis stage IV (*n*/%)	3/2	57/56	**< 0.001**
Total number of teeth at BL	Start of SPC 3486	Insertion of major restorations 1949	
Mean number of teeth at BL	26.4 ± 1.6	19.3 ± 5.0	**< 0.001**
Total number of teeth at final examination	3.317	1.544	
Years of SPC (mean ± standard deviation; minimum–maximum)	23.7 ± 2.7; (20–29)	23.8 ± 2.9; (20–29) After insertion of major restorations 19 ± 5.3; (10–29)	0.806 **< 0.001**
SPC treatment sessions	55.6 ± 16.6	58.6 ± 20.2	0.148
Fully compliant (*n*/%)	99/74	64/64	0.055
Patients with tooth/implant loss (*n*/%)	**69/52**	**73/73**	0.002
Total tooth loss	169	405	
Average tooth loss Minimum/maximum	1.3 ± 1.8 0/8	4.0 ± 4.9 0/20	**< 0.001**
Average tooth loss/year of SPC	0.05 ± 0.08	0.24 ± 0.33	**< 0.001**

Abbreviations: BL, baseline; SPC, supportive periodontal care.

**TABLE 2:** Tooth loss in patients without and with different major partial restaurations (bold: *p* < 0.001).

	Without major restorations	RPD retained with clasps	RPD (double crowns)	Fixed partial dentures	Implants	*p*
Number of patients	132	12	12	50	27	
Men (*n*/%)	104/79	8/67	11/92	37/74	23/85	**< 0.001**
Age at BL (years)	42.6 ± 10.3*	49 ± 5.3	47.6 ± 5.1	46.1 ± 6.9**	43.9 ± 1.0*	**< 0.001**
Smoking BL (*n*/%)						
Non‐smokers	56/42	3/25	1/8	15/30	6/22	0.016
Former smokers	25/19	3/25	2/17	9/18	5/19	
1–10 cigarettes/day	33/25	1/8	2/17	11/22	6/22	
< 10 cigarettes/day	18/14	5/42	7/58	15/30	10/37	
Diabetes (*n*/%)	16/11	3/25	3/25	6/12	1/4	0.250
School education at BL (*n*/%)						
Middle school	1/1	1/8	0/0	1/2	1/4	0.134
Middle school + 2 years	35/27	5/42	5/42	9/18	6/22	
High school	35/27	3/25	6/50	21/42	9/33	
Post‐secondary education	61/45	3/25	1/8	19/38	11/41	
Periodontitis stage III (*n*/%)	129/98	2/17	3/25	25/50	14/52	**< 0.001**
Periodontitis stage IV (*n*/%)	3/2	10/83	9/75	25/50	13/48	**< 0.001**
Total number of teeth at BL	Start of SPC	Insertion of major restorations				
	3486	219	206	1029	495	
Mean number of teeth at BL	26.4 ± 1.63*	18.3 ± 2.5	16.5 ± 17.2	20.6 ± 4.5**	18.3 ± 6.5	**< 0.001**
Total number of teeth at final examination	3317	134	127	838	445	
Years of SPC (mean ± standard deviation; minimum–maximum)	23.7 ± 2.7; (20–29)	23.2 ± 3.1; (20–28)	24.2 ± 2.6; (20–28)	23.8 ± 3; (20–29)	23.8 ± 2.9; (20–29)	
		After insertion of major restorations				
		18.9 ± 4.9; (13–26)	21.5 ± 4.6; (12–28)	20.5 ± 5; (10–29)	15.3 ± 4.5; (10–25)	**< 0.001**
SPC treatment sessions	55.6 ± 16.6*	56.3 ± 17.4**	67.6 ± 17.1**	61.6 ± 19.4	50.1 ± 22***	0.127
Fully compliant (*n*/%)	99/74	7/58	9/75	35/70	13/48	0.071
Patients with tooth/implant loss (*n*/%)	**69/52**	**11/92**	**9/75**	**39/78**	**14/52**	0.002
Total tooth loss	169	85	79	191	50	
Average tooth loss Minimum/maximum	1.3 ± 1.8*	7.1 ± 6.3 0/17	6.6 ± 7 0/18	3.8 ± 4.3 0/20	1.9 ± 2.8* 0/9	**< 0.001**
Average tooth loss/year of SPC	0.05 ± 0.08*	0.4 ± 0.39	0.35 ± 0.42	0.23 ± 0.33**	0.15 ± 0.22*	**< 0.001**

Abbreviations: BL, baseline; RPD, removable partial denture; SPC, supportive periodontal care.

* *p* < 0.001 to all other groups.

** *p* < 0.001 to RPD.

*** *p* < 0.001 to FPD.

A total of 163 patients were completely adherent during 23.6 ± 2.7 years of SPC and had an annual TL rate of 0.12 ± 0.21. Among these, 125 stage III periodontitis patients had an annual TL rate of 0.09 ± 0.18, and 38 stage IV periodontitis patients had an annual TL rate of 0.20 ± 0.26. Seventy patients were partially adherent and had an annual TL rate of 0.17 ± 0.30 (48 stage III patients 0.14 ± 0.31; 22 stage IV patients 0.24 ± 0.28).

At baseline, 81 patients were non‐smokers, 44 were former smokers, 53 were smokers with a daily consumption of 1–10 cigarettes (S1) and 55 were smokers with ≥ 10 cigarettes/day (S2) (Table 1). Of a total of 108 baseline smokers, 39 became non‐smokers during SPC (S1: 32; S2: 7). A further 13 of S2 reduced their tobacco consumption to the S1 level. The annual TL rate was 0.27 ± 0.32 for the 55 baseline S2 patients and 0.07 ± 0.12 for the 53 baseline S1 patients, 0.10 ± 0.14 for 105 baseline former smokers and 0.10 ± 0.25 for 81 baseline non‐smokers. Regarding socioeconomic status or treatment duration, there were no differences between both groups. However, the duration of SPC after insertion of PD was significantly shorter than for controls (*p* < 0.001; Table 1). Twenty‐nine patients (12.4%) had type II diabetes mellitus at baseline or developed it during treatment.

Negative binomial regressions identified any type of major PD (rate ratio 3.08; *p* < 0.001), age (rate ratio 1.06 per year; *p* < 0.001) and S2 (rate ratio 2.54; *p* < 0.001) as factors significantly contributing to TL in general (Table 3). Patients with RPDCs had a 37% higher risk (*p* = 0.001), with RPDDs 35% higher (*p* = 0.002), and those with FPDs a 9% higher risk (*p* < 0.001) for TL than patients without PD. Patients with iFPD had a 50% lower risk. However, this association was not significant (Table 4).

**TABLE 3:** Negative binomial regression. Dependent variable: tooth loss during supportive periodontal care/insertion of major restorations (bold: *p* < 0.001).

Variable	Estimate	*p*	Rate ratio	Lower 95% confidence interval	Upper 95% confidence interval
Major restoration	**1.125**	**< 0.001**	**3.08**	**2.08**	**4.57**
Age (1 year)	**0.061**	**< 0.001**	**1.06**	**1.04**	**1.09**
Female sex	0.076	0.711	1.08	0.72	1.61
Strict compliance	−0.218	0.236	0.80	0.56	1.15
SPC years	0.014	0.476	1.01	0.98	1.06
Non‐smoker (reference)					
Former smoker	−0.124	0.628	0.88	0.54	1.46
≤ 10 cigarettes/day	−0.200	0.412	0.82	0.51	1.32
> 10 cigarettes/day	**0.932**	**< 0.001**	**2.54**	**1.61**	**3.99**
Middle school (reference)					
Middle school +2 years	−0.081	0.897	0.92	0.27	3.16
High school	−0.357	0.569	0.70	0.21	2.39
Post‐secondary education	−0.660	0.294	0.52	0.15	1.77

**TABLE 4:** Negative binomial regression (bold: *p* < 0.001).

Variable	Estimate	*p*	Rate ratio	Lower 95% confidence interval	Upper 95% confidence interval
No major restoration (reference)					
RPDC	1.374	0.001	3.95	1.95	8.01
RPDD	1.346	0.002	3.84	1.90	7.78
FDP	**1.092**	**< 0.001**	**2.98**	**1.95**	**4.55**
iFPD	0.501	0.134	1.65	0.86	3.18
Age (1 year)	**0.059**	**< 0.001**	**1.06**	**1.04**	**1.09**
Female sex	0.050	0.806	1.05	0.71	1.57
Strict compliance	−0.239	0.190	0.79	0.55	1.23
SPC years	−0.002	0.921	1.00	0.96	1.04
Non‐smoker (reference)					
Former smoker	−0.139	0.582	0.87	0.53	1.43
≤ 10 cigarettes/day	−0.116	0.625	0.89	0.56	1.42
> 10 cigarettes/day	**0.860**	**< 0.001**	**2.36**	**1.50**	**3.73**
Middle school (reference)					
Middle school +2 years	−0.211	0.732	0.81	0.24	2.72
High school	−0.466	0.451	0.62	0.19	2.11
Post‐secondary education	−0.731	0.237	0.48	0.14	1.62

*Note*: Dependent variable: tooth loss during supportive periodontal care/after insertion of major restorations. Bold: *p* < 0.001.

Abbreviations: FPD, fixed partial denture; iFPD, FPD on implants; RPD, removable partial denture; RPDC, RPD with clasps; RPDD, RPD with double crowns.

After 10 years, in 75% of patients 67% of the RPDCs; in 80% of patients 75% of RPDDs; in 90% of patients 93% of FPDs; and in 76% of patients 83% of iFPDs were functional (Table 5). In 28% of patients, 14% of implants were lost because of peri‐implantitis. Logistic regression identified RPDC, RPDD and erratic compliance as independent factors to be associated with prosthesis retention at 10 years (Table 6).

**TABLE 5:** Restoration‐related and patient‐related 10‐year survival rates of different types of dental restorations.

	Restoration‐related	Patient‐related
Removable partial denture (clasps)	67%	75%
Removable partial denture (double crowns)	75%	80%
Fixed partial dentures ≥ 5 units	93%	90%
Implant‐supported fixed partial dentures	83%	76%

**TABLE 6:** Partial denture survival after 10 years of function (logistic regression analysis). Fixed partial dentures (FPD) with at least 5 units (abutments and pontics); removable metal‐based partial dentures (RPD) retained with clasps (RPDC); RPD retained with double crowns (RPDD); FPD on implants (iFPD).

Dependent variable: partial denture survival; *n* = 101; χ^2^ = 21.744, *p* = 0.016					
Variable	Estimate	*p*	Odds ratio	Lower 95% confidence interval	Upper 95% confidence interval
iFPD (reference)					
RPDC	4.387	0.013	80.41	2.08	2527.80
RPDD	4.155	0.016	63.78	1.04	1869.89
FPD	2.030	0.149	7.61	0.49	119.61
Age	−0.047	0.961	0.956	0.84	1.09
Female sex	−38.408	1.000	0	0	0
Strict compliance	−1.986	0.01	0.14	0.02	0.83
Number SPC after insertion	0.043	0.109	1.00	0.91	1.01
Smoking	−0.124	0.628	1.33	0.61	2.93
Diabetes	0.565	0.582	1.76	0.24	13.18

In 27 patients, 218 implants were inserted. Thirty of these implants were lost due to peri‐implantitis in 15 patients. Five of the stage III patients lost eight of their implants. Seven stage IV patients received 119 implants and lost 22 of them due to peri‐implantitis.

## 4 Discussion

### 4.1 Annual Tooth Loss

For this retrospective cohort study, tooth loss was chosen as the main outcome variable. A total of 233 patients with generalised stage III/IV periodontitis were treated over 21.7 ± 2.7 years: 12 with 21 RPDCs (TL/year 0.4 ± 0.39), 12 with 20 RPDDs (0.35 ± 0.42), 50 with 80 ≥ 5‐unit FPD (0.23 ± 0.33) and 27 with 218 implants (0.15 ± 0.22). One‐hundred and thirty‐two patients without FPD ≥ 5 units or RPD or iFPD served as control. The control group without major PD exhibited the lowest rate of TL (0.05 ± 0.08; *p* < 0.001). Multivariate analyses revealed age (*p* < 0.001), smoking > 10 cigarettes/day (*p* < 0.001) and RPD (*p* = 0.002)/FPD (*p* < 0.001) to be significantly associated with TL. Compared to that in control, the risk of TL of iFPD was reduced by 50%. However, this association was not significant (*p* = 0.134).

For RPDs, evidence on the risk of TL in periodontally treated patients between 1966 and 2020 is based on nine studies (Gotfredsen et al. 2022). Three studies indicated an increased risk of TL for patients with RPDs compared with no treatment or FPDs (Muller et al. 2013; Cabanilla et al. 2009). Walter et al. (2018) did not find any increased risk for TL in patients with RPDs. Three studies indicated that TL was more related to confounding factors than the prosthetic treatment (Isidor and Budtz‐Jorgensen 1990; Muller et al. 2013; Walter et al. 2018). It has been claimed that the risk of TL in patients with periodontitis increases when using RPDs (Leung et al. 2006; Pretzl et al. 2008). Indeed, higher TL was reported in the RPD groups compared to the control group or FPD group in two cohort studies (Isidor and Budtz‐Jorgensen 1990; Cabanilla et al. 2009).

The high number of current smokers, patients with diabetes and the advanced age at initiation of therapy in this study may explain the higher rates of TL in RPD patients compared with other studies (Chambrone and Chambrone 2006; Leung et al. 2006; Faggion Jr. et al. 2007; Eickholz et al. 2008; Pretzl et al. 2008; Baumer et al. 2011). In multivariate analyses, we observed that PD is associated with a higher risk for tooth loss than control and iFPD. Interestingly, iFPD was less associated with TL than conventional FPDs. This may be due to the fact that iFPDs do not require teeth as abutments. Use of periodontally compromised but successfully treated teeth may lead to overload or endodontic complications and early TL (Pretzl et al. 2008). Initial diagnosis was not identified as a significant factor in TL.

The annual TL rate in 12 patients treated in this study with RPDCs was 0.4 ± 0.39, and in 12 with RPDDs it was 0.35 ± 0.42. As this group of patients was significantly older and encompassed more smokers, this may be the reason why removable and expandable dentures were recommended by the dentist and chosen by the patient. Negative binomial regression analysis confirms this interpretation. In the 50 patients who only received FPDs, the annual TL was 0.23 ± 0.33, and in the 27 patients with iFPDs it was 0.15 ± 0.22, that is, below the rate for the German population (regardless of the severity of periodontitis and regardless of treatment) between the age groups 35 and 44 and 65 and 74 (IDZ V: 0.30 teeth/year) (Stark and Nitschke 2016; Nitschke and Stark 2016).

Within this cohort, 163 (70%) patients were classified as completely adherent concerning the perceived SPC intervals over 21.7 years. This aligns with a retrospective study with 76% adherence (Muller et al. 2013) but differs from other studies which also included non‐adherent patients (Wood et al. 1989; Checchi et al. 2002; Eickholz et al. 2008). By excluding non‐adherent patients, we expected less TL and less influence of other dental practitioners. Regular SPC is associated with less TL than irregular SPC (Saminsky et al. 2015; Eickholz et al. 2008). Interestingly, strict or partial adherence to SPC made no difference regarding TL in this study. However, partial adherence was associated with 10‐year survival of partial dentures, indicating that survival, particularly of RPDs, is influenced by different factors than survival of teeth.

### 4.2 10‐Year Survival Rates of Dentures and Implants

After 10 years, in 75% of patients 67% of the RPDCs; in 80% of patients 75% of RPDDs; in 90% of patients 93% of FPDs; and in 76% of patients 83% of implant‐supported FPDs were functional. In 28% of patients, 14% of implants were lost due to peri‐implantitis.

The level of function and design of restorations should be compatible with case stability over time. In partially edentulous stage IV periodontitis patients (case type 3 and 4) with tooth‐delimited edentulous spaces, different options (i.e., FPDs, iFPDs, RPDs or no PD) may be considered (Montero et al. 2022; Gotfredsen et al. 2022; Carra et al. 2022). Although patients without PD or with iFPDs were associated with less TL during SPC/after insertion of PD, RPDs exhibited better 10‐year survival than iFPDs. This may be a paradox at first sight. However, whereas implant or tooth loss will compromise the whole FPD and is likely to result in failure, RPDs are more resilient to the loss of single‐abutment teeth.

## 5 Limitations

The present study has limitations. It is an observational study with a certain risk of selection bias. Further, the number of patients in the different treatment groups was different. In addition, the reason for tooth loss was often unclear. Thus, some teeth may have been lost because of caries or trauma. Further, this study investigated a special patient group: members of the German Armed Forces (Bundeswehr) of different rank groups periodontally treated in a military periodontal specialist centre. With the start of treatment in 1993, this explains that females are underrepresented, 21.7% (Muller et al. 2013). Soldiers undergo mandatory dental examinations by the military to determine their dental fitness, and dental treatment is covered to a higher extent than for civilians. Dental treatment, on the other hand, is compulsory only for deployment. The results cannot be extrapolated to the whole of German population.

## 6 Conclusions

Within the limitations of this retrospective cohort study, the following conclusions may be drawn:

– Stage III/IV periodontitis patients with teeth replaced by RPDC, RPDD or FDP have a higher long‐term TL rate than controls.
– There seems to be no difference regarding long‐term TL rate between control and iFPD.
– Age and heavy smoking (> 10 cigarettes/day) are significantly associated with TL.
– More than 80% of PD survived at least 10 years.

## Author Contributions

All authors contributed substantially to the interpretation of the data for the work. They contributed to drafting and critically revising the manuscript, gave their final approval of the version to be published and agreed to be accountable for all aspects of the work. In particular, F.W. contributed to the conceptualisation, formal analysis, investigation, and methodology. T.E. contributed to the conceptualisation, investigation and methodology. N.L. contributed to formal analysis and methodology. P.E. contributed to the conceptualisation, formal analysis, investigation and methodology.

## Ethics Statement

The study was performed in full accordance with ethical principles according to the guidelines of the Helsinki Declaration. Its retrospective part (1993–2013) was approved by the Institutional Review Board for Human Studies of the Medical Faculty of the Johann Wolfgang Goethe‐University Frankfurt/Main (Application # 53/11). The prospective part of the study (2014–2023) was conducted at the Bundeswehr Medical Service Academy, and approved by the Regional Ethics Review of the State Chamber of Physicians of Rhineland‐Palatinate in Germany (837.486.13/9171‐F; March 28, 2014).

## Conflicts of Interest

The authors declare no conflicts of interest. The funders had no role in the study's design, the collection, analysis, or interpretation of data, the writing of the manuscript, or the decision to publish the results. The opinions expressed in this article are those of the authors. They cannot be construed as reflecting the views of the Bundeswehr Medical Service, the Bundeswehr at large, or the German Ministry of Defence.

## Data Availability

Research data are not shared.

## References

[jcpe70036-bib-0001] Baumer, A. , B. Pretzl , R. Cosgarea , et al. 2011. “Tooth Loss in Aggressive Periodontitis After Active Periodontal Therapy: Patient‐Related and Tooth‐Related Prognostic Factors.” Journal of Clinical Periodontology 38: 644–651. 10.1111/j.1600-051X.2011.01733.x. 21564157

[jcpe70036-bib-0002] Billings, M. , B. Holtfreter , P. N. Papapanou , G. L. Mitnik , T. Kocher , and B. A. Dye . 2018. “Age‐Dependent Distribution of Periodontitis in Two Countries: Findings From NHANES 2009 to 2014 and SHIP‐TREND 2008 to 2012.” Journal of Periodontology 89, no. Suppl 1: S140–S158. 10.1002/JPER.17-0670. 29926940 PMC6028062

[jcpe70036-bib-0003] Cabanilla, L. L. , A. L. Neely , and F. Hernandez . 2009. “The Relationship Between Periodontal Diagnosis and Prognosis and the Survival of Prosthodontic Abutments: A Retrospective Study.” Quintessence International 40: 821–831. 19898713

[jcpe70036-bib-0004] Carra, M. C. , H. Range , P. J. Swerts , K. Tuand , K. Vandamme , and P. Bouchard . 2022. “Effectiveness of Implant‐Supported Fixed Partial Denture in Patients With History of Periodontitis: A Systematic Review and Meta‐Analysis.” Journal of Clinical Periodontology 49, no. Suppl 24: 208–223. 10.1111/jcpe.13481. 34775625

[jcpe70036-bib-0005] Carral, C. , J. Flores‐Guillen , E. Figuero , et al. 2021. “Peri‐Implant Radiographic Bone Level and Associated Factors in Spain.” Journal of Clinical Periodontology 48: 805–815. 10.1111/jcpe.13437. 33527462

[jcpe70036-bib-0006] Chambrone, L. A. , and L. Chambrone . 2006. “Tooth Loss in Well‐Maintained Patients With Chronic Periodontitis During Long‐Term Supportive Therapy in Brazil.” Journal of Clinical Periodontology 33: 759–764. 10.1111/j.1600-051X.2006.00972.x. 16899027

[jcpe70036-bib-0007] Checchi, L. , M. Montevecchi , M. R. Gatto , and L. Trombelli . 2002. “Retrospective Study of Tooth Loss in 92 Treated Periodontal Patients.” Journal of Clinical Periodontology 29: 651–656. 12354091 10.1034/j.1600-051x.2002.290710.x

[jcpe70036-bib-0008] Demetriou, N. , A. Tsami‐Pandi , and A. Parashis . 1995. “Compliance With Supportive Periodontal Treatment in Private Periodontal Practice. A 14‐Year Retrospective Study.” Journal of Periodontology 66: 145–149. 10.1902/jop.1995.66.2.145. 7730966

[jcpe70036-bib-0009] Donos, N. , L. Andre Mezzomo , N. Mardas , M. Goldoni , and E. Calciolari . 2022. “Efficacy of Tooth‐Supported Compared to Implant‐Supported Full‐Arch Removable Prostheses in Patients With Terminal Dentition. A Systematic Review.” Journal of Clinical Periodontology 49, no. Suppl 24: 224–247. 10.1111/jcpe.13477. 34775624

[jcpe70036-bib-0010] Eger, T. , R. Thierbach , S. Hartung , G. Boros , and A. Kashta . 2013. “Effektive langfristige unterstützende Parodontitistherapie.” Parodontologie 24: 243–253.

[jcpe70036-bib-0011] Eickholz, P. , J. Kaltschmitt , J. Berbig , P. Reitmeir , and B. Pretzl . 2008. “Tooth Loss After Active Periodontal Therapy. 1: Patient‐Related Factors for Risk, Prognosis, and Quality of Outcome.” Journal of Clinical Periodontology 35: 165–174. 10.1111/j.1600-051X.2007.01184.x. 18199150

[jcpe70036-bib-0012] Faggion, C. M., Jr. , G. Petersilka , D. E. Lange , J. Gerss , and T. F. Flemmig . 2007. “Prognostic Model for Tooth Survival in Patients Treated for Periodontitis.” Journal of Clinical Periodontology 34: 226–231. 10.1111/j.1600-051X.2006.01045.x. 17257157

[jcpe70036-bib-0013] Fardal, O. , and G. J. Linden . 2008. “Tooth Loss and Implant Outcomes in Patients Refractory to Treatment in a Periodontal Practice.” Journal of Clinical Periodontology 35: 733–738. 10.1111/j.1600-051X.2008.01247.x. 18498381

[jcpe70036-bib-0014] Gennai, S. , R. Izzetti , M. C. Pioli , L. Music , and F. Graziani . 2022. “Impact of Rehabilitation Versus Edentulism on Systemic Health and Quality of Life in Patients Affected by Periodontitis: A Systematic Review and Meta‐Analysis.” Journal of Clinical Periodontology 49, no. Suppl 24: 328–358. 10.1111/jcpe.13526. 34761419

[jcpe70036-bib-0015] Gotfredsen, K. , S. Rimborg , and A. Stavropoulos . 2022. “Efficacy and Risks of Removable Partial Prosthesis in Periodontitis Patients: A Systematic Review.” Journal of Clinical Periodontology 49, no. Suppl 24: 167–181. 10.1111/jcpe.13519. 34761421

[jcpe70036-bib-0016] Hamp, S. E. , S. Nyman , and J. Lindhe . 1975. “Periodontal Treatment of Multirooted Teeth. Results After 5 Years.” Journal of Clinical Periodontology 2: 126–135. 1058213 10.1111/j.1600-051x.1975.tb01734.x

[jcpe70036-bib-0017] Herrera, D. , M. Sanz , M. Kebschull , et al. 2022. “Treatment of Stage IV Periodontitis: The EFP S3 Level Clinical Practice Guideline.” Journal of Clinical Periodontology 49, no. Suppl 24: 4–71. 10.1111/jcpe.13639. 35688447

[jcpe70036-bib-0018] Isidor, F. , and E. Budtz‐Jorgensen . 1990. “Periodontal Conditions Following Treatment With Distally Extending Cantilever Bridges or Removable Partial Dentures in Elderly Patients. A 5‐Year Study.” Journal of Periodontology 61: 21–26. 10.1902/jop.1990.61.1.21. 2313518

[jcpe70036-bib-0019] Lange, D. E. , H. C. Plagmann , A. Eenboom , and A. Promesberger . 1977. “Klinische Bewertungsverfahren zur Objektivierung der Mundhygiene.” Deutsche zahnärztliche Zeitschrift 32: 44–47. 264444

[jcpe70036-bib-0020] Leung, W. K. , D. K. Ng , L. Jin , and E. F. Corbet . 2006. “Tooth Loss in Treated Periodontitis Patients Responsible for Their Supportive Care Arrangements.” Journal of Clinical Periodontology 33: 265–275. 10.1111/j.1600-051X.2006.00903.x. 16553635

[jcpe70036-bib-0021] Lindhe, J. , and S. Nyman . 1977. “The Role of Occlusion in Periodontal Disease and the Biological Rationale for Splinting in Treatment of Periodontitis.” Oral Sciences Reviews 10: 11–43. 335305

[jcpe70036-bib-0022] Montero, E. , A. Molina , D. Palombo , B. Moron , G. Pradies , and I. Sanz‐Sanchez . 2022. “Efficacy and Risks of Tooth‐Supported Prostheses in the Treatment of Partially Edentulous Patients With Stage IV Periodontitis. A Systematic Review and Meta‐Analysis.” Journal of Clinical Periodontology 49, no. Suppl 24: 182–207. 10.1111/jcpe.13482. 34786742

[jcpe70036-bib-0023] Muller, S. , P. Eickholz , P. Reitmeir , and T. Eger . 2013. “Long‐Term Tooth Loss in Periodontally Compromised but Treated Patients According to the Type of Prosthodontic Treatment. A Retrospective Study.” Journal of Oral Rehabilitation 40: 358–367. 10.1111/joor.12035. 23362962

[jcpe70036-bib-0024] Nitschke, I. , and H. Stark . 2016. “Krankheits‐ und Versorgungsprävalenzen bei Jüngeren Senioren (65‐ bis 74‐Jährige). Zahnverlust und prothetische Versorgung.” In Fünfte Deutsche Mundgesundheitsstudie. DMS V, edited by A. R. Jordan and W. Micheelis , 416–451. Deutscher Ärzteverlag.

[jcpe70036-bib-0025] Papapanou, P. N. , M. Sanz , N. Buduneli , et al. 2018. “Periodontitis: Consensus Report of Workgroup 2 of the 2017 World Workshop on the Classification of Periodontal and Peri‐Implant Diseases and Conditions.” Journal of Clinical Periodontology 45, no. Suppl 20: S162–S170. 10.1111/jcpe.12946. 29926490

[jcpe70036-bib-0026] Pretzl, B. , J. Kaltschmitt , T. S. Kim , P. Reitmeir , and P. Eickholz . 2008. “Tooth Loss After Active Periodontal Therapy. 2: Tooth‐Related Factors.” Journal of Clinical Periodontology 35: 175–182. 10.1111/j.1600-051X.2007.01182.x. 18199151

[jcpe70036-bib-0027] Saminsky, M. , M. Halperin‐Sternfeld , E. E. Machtei , and J. Horwitz . 2015. “Variables Affecting Tooth Survival and Changes in Probing Depth: A Long‐Term Follow‐Up of Periodontitis Patients.” Journal of Clinical Periodontology 42: 513–519. 10.1111/jcpe.12419. 25970318

[jcpe70036-bib-0028] Sanz, M. , D. Herrera , M. Kebschull , et al. 2020. “Treatment of Stage I‐III Periodontitis‐The EFP S3 Level Clinical Practice Guideline.” Journal of Clinical Periodontology 47, no. Suppl 22: 4–60. 10.1111/jcpe.13290. 32383274 PMC7891343

[jcpe70036-bib-0029] Schwarz, F. , J. Derks , A. Monje , and H. L. Wang . 2018. “Peri‐implantitis.” Journal of Clinical Periodontology 45, no. Suppl 20: S246–S266. 10.1111/jcpe.12954. 29926484

[jcpe70036-bib-0030] Stark, H. , and I. Nitschke . 2016. “Krankheits‐ und Versorgungsprävalenzen bei Jüngeren Erwachsenen (35‐ bis 44‐Jährige). Zahnverlust und prothetische Versorgung.” In Fünfte Deutsche Mundgesundheitsstudie DMS V, edited by A. R. Jordan and W. Micheelis , 335–358. Deutscher Ärzteverlag.

[jcpe70036-bib-0031] Tonetti, M. S. , H. Greenwell , and K. S. Kornman . 2018. “Staging and Grading of Periodontitis: Framework and Proposal of a New Classification and Case Definition.” Journal of Clinical Periodontology 45, no. Suppl 20: S149–S161. 10.1111/jcpe.12945. 29926495

[jcpe70036-bib-0032] Walter, M. H. , J. Dreyhaupt , W. Hannak , et al. 2018. “The Randomized Shortened Dental Arch Study: Tooth Loss Over 10 Years.” International Journal of Prosthodontics 31: 77–84. 10.11607/ijp.5368. 29316570

[jcpe70036-bib-0033] Wood, W. R. , G. W. Greco , and W. T. McFall Jr. 1989. “Tooth Loss in Patients With Moderate Periodontitis After Treatment and Long‐Term Maintenance Care.” Journal of Periodontology 60: 516–520. 10.1902/jop.1989.60.9.516. 2677303

